# Microembolic Signals Detected with Transcranial Doppler Sonography Differ between Symptomatic and Asymptomatic Middle Cerebral Artery Stenoses in Northeast China

**DOI:** 10.1371/journal.pone.0088986

**Published:** 2014-02-14

**Authors:** Xiujuan Wu, Hongliang Zhang, Haiyu Liu, Yingqi Xing, Kangding Liu

**Affiliations:** The Neuroscience Center, Department of Neurology, The First Hospital of Jilin University, Jilin University, Changchun, China; University of Regensburg, Germany

## Abstract

Although microembolus monitoring has been widely used for ischemic cerebrovascular disease, the clinical significance of microembolic signal (MES) in asymptomatic middle cerebral artery (MCA) stenosis remains unclear. We aim to investigate the frequency of MES and the value of MES in predicting ischemic stroke secondary to asymptomatic MCA stenosis. From June 2011 to December 2012, microembolus monitoring was performed in 83 asymptomatic and 126 symptomatic subjects. By comparing the demographics and risk factors between the symptomatic and asymptomatic subjects, we found that the ratio of male sexuality and smoking history differed (101/126 vs 43/83, and 88/126 vs 38/83, respectively, *p*<0.01). The frequency of MES was significantly higher in the symptomatic group than in the asymptomatic group (49/126 vs 2/108, *p*<0.01). Specifically, the frequency of MES in the symptomatic and asymptomatic groups with mild stenosis, moderate stenosis, severe stenosis and occlusion groups was 4/18 (22.22%) vs 0/30 (0), 13/31 (41.94%) vs 1/28 (3.57%), 30/62 (48.39%) vs 1/39 (2.56%), 2/15 (13.33%) vs 0/11 (0), respectively. Except for the occlusive group, the frequency of MES is correlated with stenosis degree and symptom. Two patients in the asymptomatic group were found positive for MES, and the MES number was 1 for both. During the one-year follow-up, neither of them developed ischemic stroke. In conclusion, MES detected with TCD differs between symptomatic and asymptomatic MCA stenoses. Due to the low frequency, the value of MES as a predictor of subsequent ischemic stroke in patients with asymptomatic MCA stenosis might be limited.

## Introduction

Atherosclerotic stenosis of intracranial artery, especially the middle cerebral artery (MCA) stenosis is a common cause of stroke in Asians, which may account for up to half of ischemic cerebrovascular events in the Chinese population [1]–[4]. The possible mechanisms for cerebral infarction with intracranial artery stenosis include thrombosis leading to complete occlusion, hemodynamic compromise, artery-to-artery embolism, local branch occlusion or combinations of these factors. Transcranial Doppler sonography (TCD) is a sensitive technique for real-time detection of microembolic signals (MES) and can be used to evaluate the vulnerability of the plaque in the patients with artery stenosis. Studies of MES on symptomatic cerebral artery stenosis are extensive, including exploring the mechanism of ischemic stroke [5], evaluating the treatment efficacy [6], and predicting recurrent cerebral ischemic events or progression [7]–[9], etc. With the popularity of TCD in China, increasingly more asymptomatic patients with risk factors were found with intracranial artery stenosis, especially with MCA stenosis. In 2010, the Asymptomatic Carotid Emboli Study (ACES) [10] found that detection of MES with TCD can be used to identify patients with asymptomatic carotid stenosis who are at a higher risk of stroke and transient ischemic attack (TIA). However, there have been few reports on the frequency and clinical significance of MES in asymptomatic MCA stenosis which occurs more common in Asians [1], [4]. In a review [11] summarizing the prevalence and prognostic impact of MES in arterial sources of embolism, the frequency of MES in 220 patients with symptomatic intracranial artery stenosis was reported to be 25% while 0% of 86 asymptomatic patients (*p*<0.0001). This review [11] included the data from seven studies on MES in intracranial artery stenosis that were conducted in western countries. However, the small number of patients with stenosed MCA is a major limitation. In Wong’s pilot study [12] investigating MES in patients with MCA stenosis, no MES was found in the 20 asymptomatic MCA stenosis group. Taken together, the relationship between MES and MCA stenosis, and the value of MES detection in stroke prediction remains unclear. Therefore, we designed the current study to compare the frequency of MES in symptomatic and asymptomatic MCA stenosis, and to see whether MES can predict ischemic stroke in patients with asymptomatic MCA stenosis.

## Subjects and Methods

### Study Setting

The study was conducted in The First Hospital of Jilin University, Changchun, China. The Neuroscience Center, Department of Neurology is the largest neurology department in northeast China. The Ultrasound Laboratory in the Department of Neurology, which possesses 10 TCD and 3 carotid duplex machines, is one of the four training centers in China.

### Subjects

This study was approved by the ethics committee of The First Hospital of Jilin University. And written informed consent was obtained from all patients. From June 2011 to December 2012, a total of 15019 subjects who visited our Ultrasound Laboratory received paired examinations with TCD and carotid duplex. MCA stenosis with or without other cerebrovascular stenosis was identified. Patients who were diagnosed as asymptomatic and symptomatic MCA stenosis and who met our inclusion criteria were enrolled in the study, all of whom were Chinese. A head computed tomography scan was performed to rule out intracranial hemorrhage in all patients. Finally, TCD, carotid duplex and MES detection were performed in 209 patients.

### Inclusion Criteria and Exclusion Criteria

Patients were included in the asymptomatic MCA stenosis group if they met the following criteria: patients had no symptoms of TIA or ischemic stroke which was confirmed by CT and/or MRI, including the patients who have no symptoms but have lacunar infarction through general health examinations; MCA stenosis diagnosed by TCD and the stenotic area was M1; without any history of ischemic stroke or TIA. In the symptomatic MCA stenosis group, the inclusion criteria were as follows: acute and clear focal neurological dysfunction which were suspected due to MCA stenosis (including TIA or ischemic stroke within 3 days of onset or progression) and all the patients performed CT and/or MRI; with ipsilateral MCA-M1 stenosis diagnosed by TCD; without receiving thrombolytic therapy.

For both the asymptomatic and symptomatic group, patients with the following conditions were excluded: presence of carotid artery stenosis ipsilateral to the MCA stenosis; presence of a poor temporal acoustic window; could not consent to participate in the study, such as the patients in an agitated or confused state; presence of other potential sources of embolism, such as cardiogenic emboli and blood-borne emboli as well as any other artery borne embolic source; and patients with severe liver or renal function or cancer.

### Vascular Risk Factors

At baseline, we recorded the demographic data including sex, age, and the main stroke risk factors, including previous history of hypertension, diabetes mellitus, smoking, dyslipidemia, ischemic heart disease and drinking. Hypertension, diabetes mellitus, and ischemic heart disease were diagnosed by cardiologists and diabetologists.

### Diagnosis of MCA Stenosis and MES Detection

Both intracranial and extracranial arteries were assessed by TCD (EME-TC8080, Nicolet, Germany) and carotid Duplex (iU22, Philips, USA). TCD examinations were performed with 2 MHz probe to detect MCA-Ml, anterior cerebral artery (ACA)-Al, posterior cerebral artery (PCA) and intracranial segment of internal carotid artery (including siphon and terminal segments) through the temporal window, intracranial segment of vertebral artery (VA) and basilar artery (BA) through the pillow window, and the ophthalmic artery and the siphon segment of internal carotid artery through the eye window. The TCD criteria for diagnosis of MCA stenosis or occlusion were based on the published criteria [13]–[16]. The criteria for classification of MCA stenosis were defined by the peak systolic flow. We defined mild stenosis as a systolic peak velocity from 140 to 209 cm/s, moderate stenosis from 210 to 280 cm/s, and severe stenosis from >280 cm/s. We diagnosed occlusion of the MCA if all the basal arteries except the MCA in question were detectable or if the asymmetry index of the affected MCA was<−21% compared with the contra-lateral MCA with the hemodynamic changes of the intracranial circulation.

MES detection was performed in all the recruited patients with TCD. We conducted MES monitoring immediately after TCD examination both for the symptomatic and asymptomatic MCA stenosis patients. Two 2 MHz probes were fixed on patients’ bilateral temporal window. Depth of investigation of the affected MCA was respectively located at prestenotic and poststenotic areas, or intrastenotic and poststenotic areas. The sample volumes were chosen to be as low as possible to avoid overlap. Additionally, the distance of the two depth of the same vessel was greater than the sample volume. Emboli originating proximal to the MCA stenosis passed through the different sample volumes, while emboli exiting from the MCA stenosis would only pass the second channel, located distal to the stenosis, and would produce a typical signal but no signal in the prestenotic sample volume. Therefore, an embolic signal would not be recorded in the proximal sample volume but only in the distal one. Artifacts produce a typical pattern in both segments at the same time. The probe on the healthy side adopted the same settings with the affected side; gain and power were turned down as far as possible. In addition, all data were continuously recorded onto a 4-channel digital audio tape recorder with normal speed. The recorded data were then analyzed by two experienced observers, who were blinded to the clinical data. The number of MES during the 30-minute recording was noted. The following definitions for emboli signals were used: typical visible and audible (click, chirp, whistle) short-duration, high-intensity signal within the Doppler flow spectrum with its occurrence at random in the cardiac cycle, and an intensity increase of ≥5 dB above the background signal, and MES recorded from MCA stenoses has special characteristics of multiple frequencies [17]–[18].

### Follow-up

Patients who were MES positive with the asymptomatic MCA stenosis were followed by telephone to see whether they received antiplatelet or statin treatment regularly, and whether they experienced TIA or stroke or other general symptoms.

### Statistical Analysis

Statistical data analysis was performed with SPSS version 17.0 software (SPSS, IBM, West Grove, PA, USA). The χ^2^ tests were used for discrete variables and the student-*t* tests for contiguous variables. The logistic regression was done for the relationship for MES and the mentioned risk factors. For all statistical tests, *p*<0.05 was considered significant.

## Results

### Baseline Demographics

**Figure 1:**
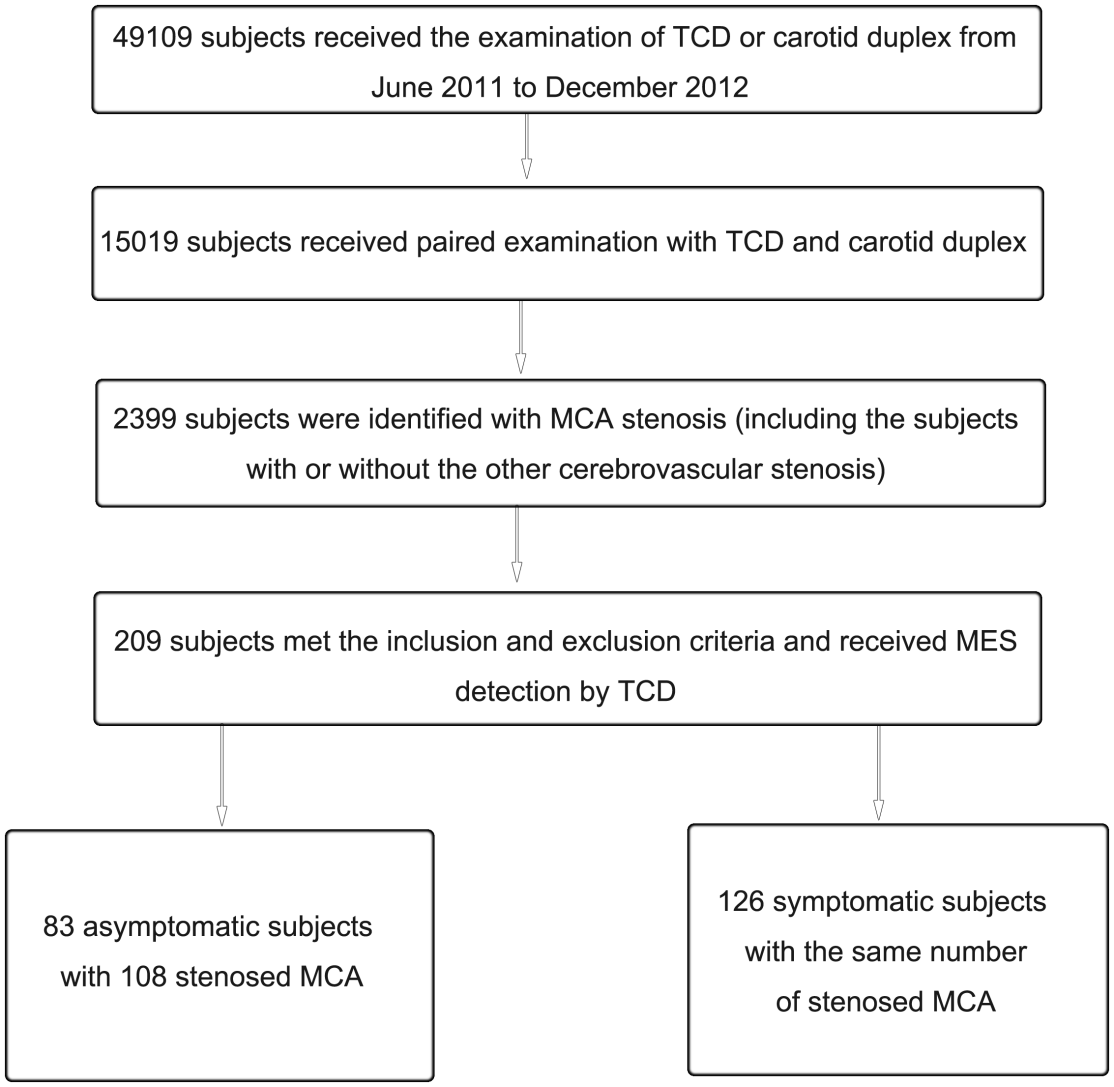
The flow chart of the study. TCD: transcranial Doppler sonography; MCA: middle cerebral artery.

**Figure 2:**
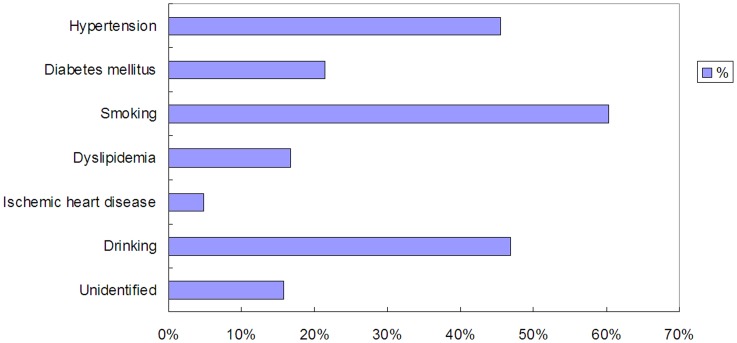
The distribution of risk factors in recruited patients. Risk factors include the history of the hypertension, diabetes mellitus, ischemic heart disease, dyslipidemia, smoking, drinking. For some patients, risk factors could not be identified.

During June 2011 to December 2012, a total of 15019 out of 49109 subjects who visited our ultrasound laboratory received paired examinations with TCD and carotid duplex. Among them, 2632 patients were found with mere intracranial artery stenosis, 1186 patients with mere extracranial artery stenosis and 1623 patients with both intracranial and extracranial artery stenosis. MCA stenosis with or without other cerebrovascular stenosis was identified in 2399 subjects (1426 males and 973 females, aged from 16 to 93 years old). Finally, 209 patients with mere MCA stenosis (83 asymptomatic patients with 108 stenosed MCA and 126 symptomatic patients with the same number of stenosed MCA) were enrolled to receive MES detection, among whom 144 were males and 65 were females, aged from 35 to 82 years old. The flow chart of the study was shown in Figure 1. The recorded baseline demographics of all recruited patients included age, sex and risk factors of ischemic stroke, i.e. the history of hypertension, diabetes mellitus, smoking, dyslipidemia, ischemic heart disease and drinking. The distribution of the risk factors of all the recruited patients was shown in Figure 2.

### Distinguishable TCD Spectrum Identifies Stenosed MCA and MES

**Figure 3:**
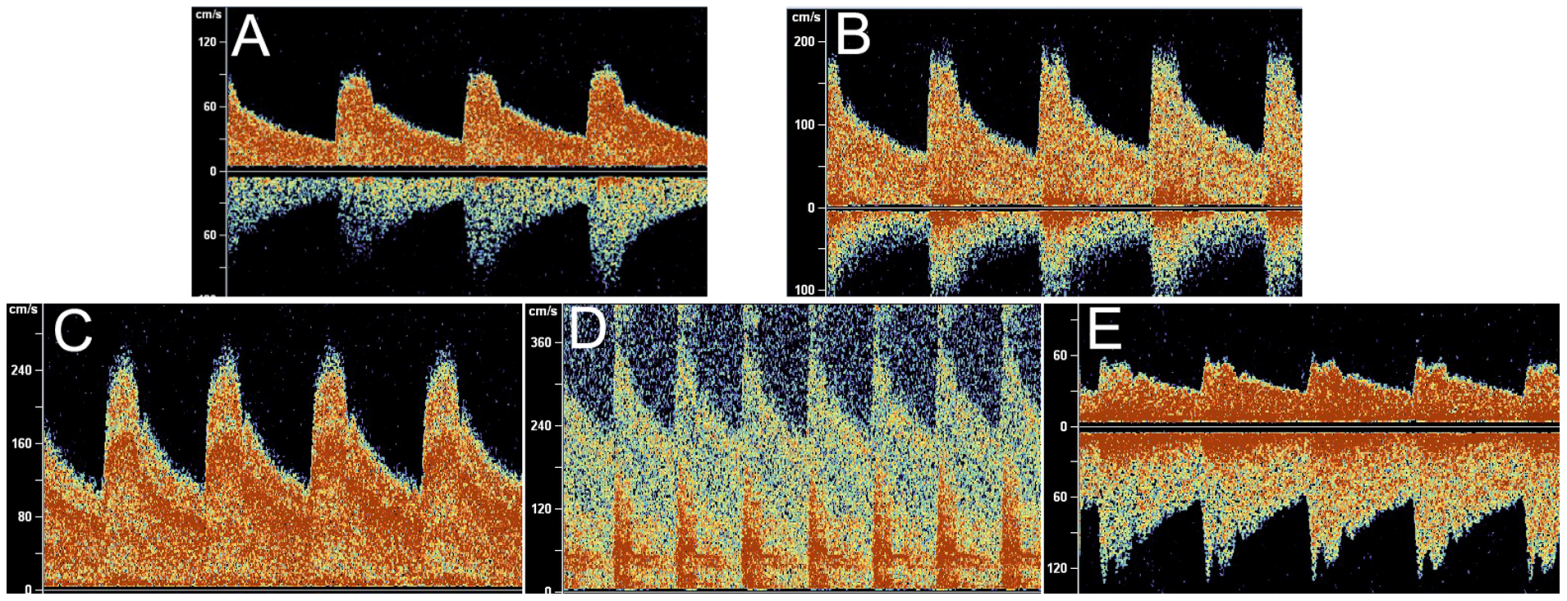
The blood flow spectrum of normal and stenosed MCA. A. Normal MCA; B. Mild MCA stenosis: mild stenosis was defined as systolic peak velocity 140 to 209 cm/s; C. Moderate MCA stenosis: moderate stenosis was defined as a systolic peak velocity from 210 to 280 cm/s; D. Severe MCA stenosis: severe stenosis was defined as a systolic peak velocity >280 cm/s; E. Occlusive of MCA: We diagnosed occlusion of the MCA if all the basal arteries except the MCA in question were detectable or if the asymmetry index of the affected MCA was<−21% compared with the contralateral MCA with the hemodynamic changes of the intracranial circulation.

All the recruited patients were performed TCD, carotid duplex and MES detection. The blood flow spectrum of the normal and stenosed MCA was shown in Figure 3. The spectrum of normal MCA was shown in Figure 3A. The stenosed MCA was grouped into mild stenosis (Figure 3B), moderate stenosis (Figure 3C), severe stenosis (Figure 3D) and occlusive group (Figure 3E) following the criteria for classification of MCA stenosis.

**Figure 4:**
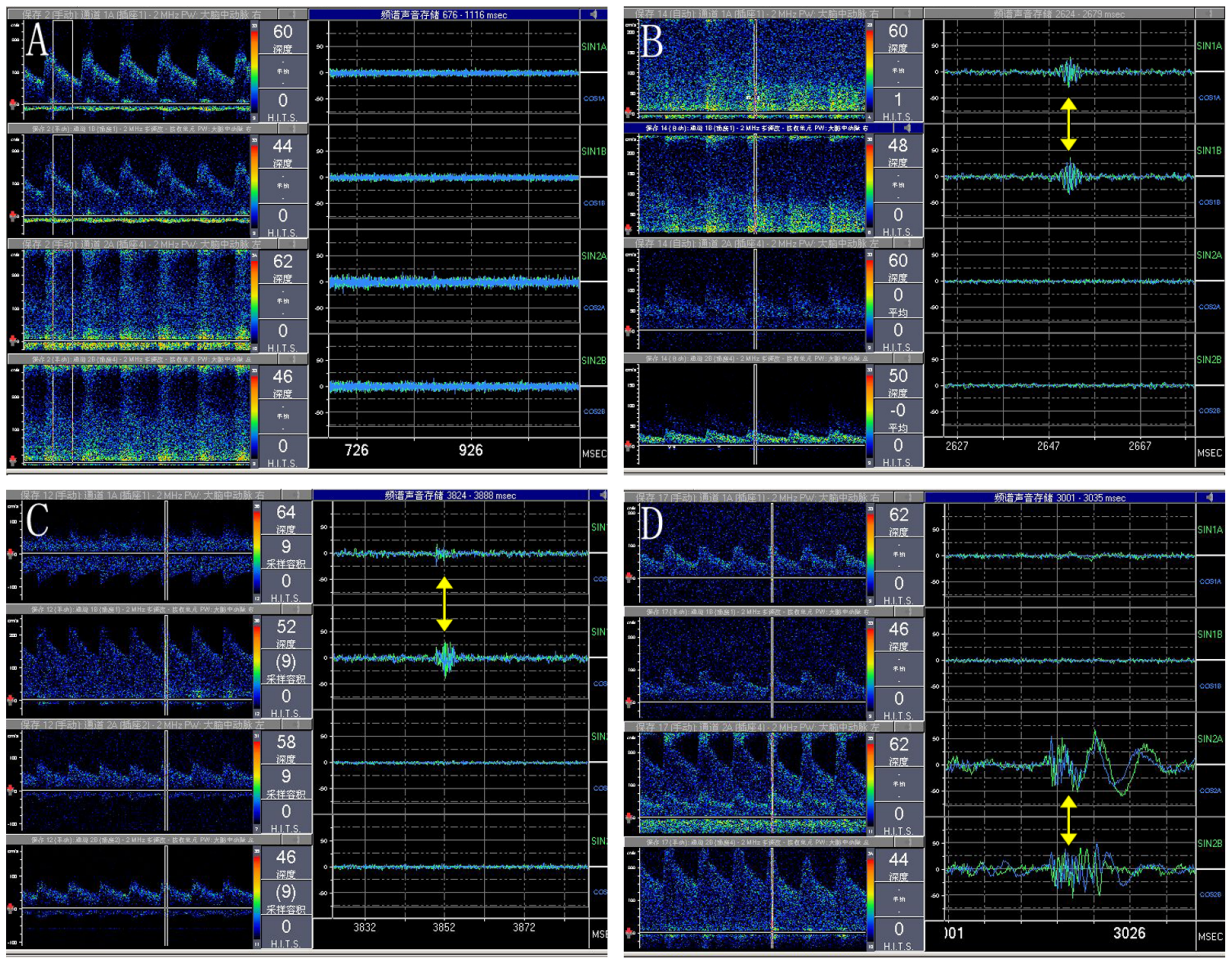
The spectrum of microembolic signal (MES) examination. A was the spectrum of the MES negative (MES−) in a patient with MCA stenosed. And the left were the spectrum of the bilateral MCA in different depths. B and C were the spectrum of MES positive (MES+) in another two patients with MCA stenosis. And we could see the typical MES (the arrow). The depth of investigation of the affected MCA was located at intrastenotic and poststenotic in B, and we could see MES in the two depth. C was located prestenotic and poststenotic areas which we could only see MES in the poststenotic areas (the distal part). D was the spectrum of an atypical MES (the arrow) in a patient with MCA stenosis.

The MES negative (MES−) and MES positive (MES+) spectrum of the stenosed MCA was shown in Figure 4. The visible short-duration, high-intensity signal within the Doppler flow spectrum with its occurrence at random in the cardiac cycle was MES+ (Figure 4B, C and D). The spectrum of the MES− was shown in Figure 4A.

### Presence of MES was not Associated with Stroke Risk Factors

**Figure 5:**
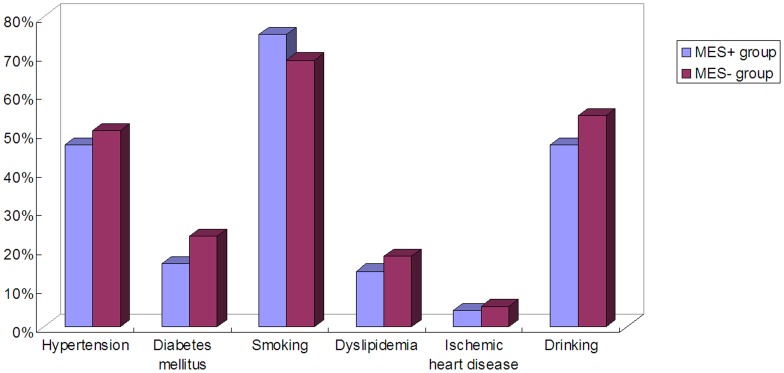
The comparison of risk factors in MES+ and MES− groups of the symptomatic MCA stenosis group. Risk factors in MES+ and MES− groups of the symptomatic MCA stenosis group were shown in the figure.

In the symptomatic group, there were 49 MES+ patients and 77 MES- patients. There was no significant difference with regard to male sexuality between the two groups (77.6% vs 81.2%, *p*>0.05), and the mean age did not differ between the MES+ group and the MES− group (52.7 vs 51.9, *p*>0.05). The risk factors (including the history of hypertension, diabetes mellitus, smoking, dyslipidemia, ischemic heart disease and drinking) of the MES+ and MES− groups in the symptomatic group were shown in Figure 5. Further, the binary logistic regression was done for the relationship for MES and the above mentioned risk factors in the symptomatic MCA stenosis group, suggesting that MES was not associated with risk factors (*p*>0.05).

### Frequency of MES is Associated with Artery Stenosis Degree and Symptom

**Figure 6:**
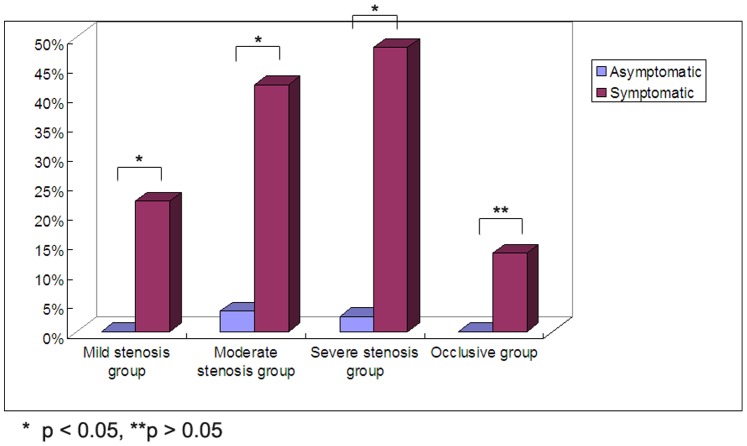
MES frequency in different grade stenosed MCA of the asymptomatic and symptomatic patients. MES frequency was compared among groups both in the asymptomatic and symptomatic patients. The frequency of MES in mild, moderate, severe stenosis and occlusive group of the symptomatic and asymptomatic groups were 4/18 (22.22%) vs 0/30 (0), 13/31 (41.94%) vs 1/28 (3.57%), 30/62 (48.39%) vs 1/39 (2.56%), 2/15 (13.33%) vs 0/11 (0), respectively. The frequency of MES in patients with severe stenosis groups was higher than those with mild stenosis and occlusion in the symptomatic MCA stenosis group with statistical difference (*p*<0.05). The frequency of MES in patients with moderate stenosis was higher than those with mild stenosis and occlusion, although there was no statistical difference (*p*>0.05). The frequency of MES did not differ between the mild stenosis group and the occlusive group (*p*>0.05). Besides, we found that except for the occlusive group, the frequency of MES in the symptomatic group was higher than the asymptomatic group in the mild, moderate and severe group, respectively (all *p*<0.05).

The frequency of MES in mild stenosis, moderate stenosis, severe stenosis and occlusive group of the symptomatic and asymptomatic groups were 4/18 (22.22%) vs 0/30 (0), 13/31 (41.94%) vs 1/28 (3.57%), 30/62 (48.39%) vs 1/39 (2.56%), 2/15 (13.33%) vs 0/11 (0), respectively (Figure 6). The frequency of MES in patients with severe stenosis was higher than those with mild stenosis and occlusion in the symptomatic group (*p*<0.05). The frequency of MES in patients with moderate stenosis was higher than those with mild stenosis and occlusion, although there was no statistical difference (*p*>0.05). But the frequency of MES did not differ between the mild stenosis group and the occlusive group (*p*>0.05). Besides, we found that except for the occlusive group, the frequency of MES in the symptomatic group was higher than asymptomatic group in the mild, moderate and severe group, respectively (all *p*<0.05).

### Frequency of MES Differed between the Symptomatic and Asymptomatic Subjects with MCA Stenosis

The clinical significance of MES in asymptomatic MCA stenosis remains unclear. By comparing the frequency of MES in symptomatic and asymptomatic groups, we found that the frequency of MES between the two groups was significantly different (49/126 vs 2/108, *p*<0.01); the MES frequency in the symptomatic group was significantly higher than the symptomatic group (Table 1). The median number of MES in symptomatic MCA stenosis group was 5 (1–65), while in the asymptomatic MCA stenosis group, it was 1 for both MES positive patients. The baseline demographics of male sexuality and smoking history between asymptomatic and symptomatic groups were different (43/83 vs 101/126, and 38/83 vs 88/126, respectively, *p*<0.01), while the other risk factors had no difference between the two groups (*p*>0.05) as shown in Table 2.

**Table 1:** MES frequency in the asymptomatic and symptomatic groups.

Number of patients	MES-positive	MES-negative	Frequency of MES	Chi-Squre	*p*-value
Asymptomatic group	2	106	2/108 (1.85%)	46.80	7.85E-12
Symptomatic group	49	77	49/126 (38.89%)		

**Table 2:** The comparison of baseline demographics of asymptomatic and symptomatic groups.

Variables	Asymptomatic group	Symptomatic group	*p*-value
Sex (M/F)	43∶40	101∶25	= 0.00001<0.01
Age	51.5±10.3	53.7±11.0	>0.05
Hypertension	33/83	62/126	>0.05
Diabetes mellitus	19/83	26/126	>0.05
Ischemic heart disease	4/83	6/126	>0.05
dyslipidemia	14/83	21/126	>0.05
Smoking history	38/83	88/126	= 0.0005<0.01
Drinking history	33/83	65/126	>0.05

### Follow-up

In the 83 asymptomatic patients with 108 stenosed MCA, there were 2 MES+ patients, whose MES number was 1. According to the telephone follow-up after one year, we found that no one developed TIA or ischemic stroke.

## Discussion

We found that the baseline demographics (including age, sex, the previous history of the hypertension, diabetes mellitus, smoking, dyslipidemia, ischemic heart disease and drinking) of the MES+ and MES- groups in the symptomatic MCA stenosis group had no difference, which suggested that the risk factors were not correlated with the frequency of MES. We further compared the baseline demographics of the asymptomatic and symptomatic groups, and found that male sexuality and smoking history of the two groups were different while the others were not. So we supposed that male and female may have an equal opportunity to develop MCA stenosis, but male and smoking patients in northeast China may develop symptomatic MCA stenosis more often.

The frequency of MES in the asymptomatic group was lower than the symptomatic group, which was in agreement with previous studies [11], [19]. Our findings demonstrated that MES was directly associated with symptom, suggesting that the plaque of the asymptomatic MCA stenosis was more stable than the symptomatic group. Except for the patients with occlusive MCA, the frequency of MES was associated with stenosis degree and symptom, which was consistent with previous studies [7]. In both the symptomatic and asymptomatic group, patients with occlusive MCA had a low frequency of MES. This might be due to the vanished blood flow and the decreased shear stress of the plaques.

MES detection by TCD is a sensitive technique for real-time detection, and it may be used to assess the vulnerability of the plaque in patients with cerebrovascular artery stenosis. Kermer and colleagues [20] observed and followed 53 patients with asymptomatic MCA stenosis and found that only one developed TIA, whose lesions were not in the blood supply region of the stenosed MCA. Ni et al [21] also found that the risk of ischemic stroke occurrence in patients with asymptomatic atherosclerotic MCA stenosis was low. The result of our study may explain the low risk of stroke occurrence in patients with asymptomatic atherosclerotic MCA stenosis of the above study. Our findings are also consistent with Li et al’s [22], [23]. They found that asymptomatic MCA stenosis presented a negative remodeling which predicted the plaque was stable. Besides, maybe the composition of plaque in the symptomatic and asymptomatic MCA stenosis is different which need further study by MRI.

Previous studies have concluded that MES could predict recurrent cerebral ischemic events in symptomatic MCA stenosis [7]. In 2010, a study of asymptomatic embolization for prediction of stroke in the Asymptomatic Carotid Emboli Study (ACES) [9] found that detection of MES on TCD can be used to identify patients with asymptomatic carotid stenosis who are at a higher risk of stroke and TIA. However, there have been few studies on MES frequency in asymptomatic MCA stenosis and on whether MES could predict the ischemic cerebrovascular events in the future in patients with asymptomatic MCA stenosis. We therefore studied whether MES could be used to predict ischemic stroke in patients with asymptomatic MCA stenosis. During the follow-up of one year in our study, the two MES+ patients in the asymptomatic group did not develop TIA or ischemic stroke. Due to the low frequency, the value of MES as a predictor of subsequent ischemic stroke in patients with asymptomatic MCA stenosis might be limited. The result is different from ACES; the reasons may be as follows: (1) we gave antiplatelet or statin treatment after asymptomatic MCA stenosis was diagnosed, which may influence the result of follow-up [6]; (2) the plaque of asymptomatic carotid stenosis and the asymptomatic MCA stenosis may be different in composition; (3) may be the asymptomatic MCA stenosis has a benign natural course expect for our treatment.

Our study had a large sample size as our Ultrasound Laboratory is the largest tertiary referral central in the northeast of China. Although the frequency of MES in the asymptomatic MCA stenosis was low, our study was strengthened by our large sample size with strict inclusion and exclusion criteria. However, our study had several limitations. Our cohort was only selected from patients examined at the Ultrasound Laboratory in The Neuroscience Center, Department of Neurology. Due to the low frequency, the value of MES as a predictor of subsequent ischemic stroke in patients with asymptomatic MCA stenosis might be limited. Besides, we only followed the MES+ patients in asymptomatic MCA stenosis group. Furthermore, we prescribed antiplatelet drugs and/or statins after diagnosing asymptomatic MCA stenosis, which may influence the result of follow-up. We hence could not exclude the possibility that the natural course of some asymptomatic MCA stenoses was benign and reversible. Finally, we diagnosed MCA stenosis with TCD and the accuracy was relatively lower than other imaging modalities like digital subtraction angiography (DSA).

In summary, MES detected with TCD differed between symptomatic and asymptomatic MCA stenoses in northeast China. Except for the patients with occlusive MCA, the frequency of MES was positively correlated with the stenosis degree and symptom, which suggested that the atherosclerotic plaque of the occlusive and asymptomatic MCA was stable. Due to the low frequency, the value of MES as a predictor of subsequent ischemic stroke in patients with asymptomatic MCA stenosis might be limited. In further study, we need to enlarge the sample size and extend the follow-up for both MES positive and negative patients with asymptomatic MCA stenosis.
